# BPA Disrupts Hepatic Lipid and Carbohydrate Metabolism in Female Zebrafish: Protective Effects of Probiotics Revealed by FTIRI and Lipidomics

**DOI:** 10.3390/ijms27072978

**Published:** 2026-03-25

**Authors:** Christian Giommi, Chiara Santoni, Fabrizia Carli, Amalia Gastaldelli, Francesca Maradonna, Hamid R. Habibi, Elisabetta Giorgini, Oliana Carnevali

**Affiliations:** 1 Department of Life and Environmental Sciences, Università Politecnica delle Marche, Via Brecce Bianche, 60131 Ancona, Italy; c.giommi@staff.univpm.it (C.G.); c.santoni@pm.univpm.it (C.S.); e.giorgini@staff.univpm.it (E.G.); 2 INBB—Biostructures and Biosystems National Institute, 00136 Roma, Italy; 3 Cardiometabolic Risk Laboratory, Institute of Clinical Physiology (IFC), National Research Council (CNR), 56124 Pisa, Italy; fabrizia.carli@cnr.it (F.C.); amalia.gastaldelli@cnr.it (A.G.); 4 Department of Biological Sciences, University of Calgary, Calgary, AB T2N 1N4, Canada; habibi@ucalgary.ca

**Keywords:** bisphenol A, SLAB51, *Danio rerio*, biochemical alterations, Fourier Transform Infrared Imaging spectroscopy, fatty acid metabolism

## Abstract

Bisphenol A (BPA) is a widespread endocrine disruptor that interferes with metabolism in humans and animals by inducing oxidative stress, lipid peroxidation, and cell death. Probiotics, conversely, have shown potential in promoting host health and reducing the toxicity of endocrine-disrupting chemicals (EDCs). This study examined whether sub-chronic BPA exposure disrupts hepatic lipid metabolism in female zebrafish (*Danio rerio*), and whether co-administration of probiotics mitigates these effects. Adult females were exposed for 28 days to the following treatments: 10 µg/L BPA via water (BPA); 10^9^ CFU/g body weight/day of probiotic formulation (P); and both treatments (BPA+P). An untreated group served as a control (CTRL). Hepatic lipid composition was analyzed using UHPLC-QTOF-MS, while liver sections were investigated by Fourier Transform Infrared Imaging (FTIRI) spectroscopy. BPA exposure decreased 14 unsaturated triacylglycerols and lysophosphatidylcholine 18:0, suggesting steatosis onset and inflammation, while in the group exposed to BPA+P, the decrease was limited to 8 triacylglycerols and the reduction in lysophosphatidylcholine 18:0 was prevented. Analyses of pooled liver samples precluded modeling tank-level effects; thus, the results are interpreted as semi-quantitative. Partial least square discriminant analysis built on the comparison of all groups together confirmed an intermediate phenotype for BPA+P fish between BPA and P groups. The observed beneficial role of probiotics in counteracting BPA-related metabolic disturbances was also supported by FTIRI, evidencing the ability to mitigate the effects of BPA on lipid and glycosylated compound metabolism. These findings highlight the potential of probiotic supplementation as a practical and accessible strategy to mitigate BPA-induced metabolic disturbances, contributing to the development of mitigating approaches against environmental contaminant-related liver dysfunction.

## 1. Introduction

Plastic-derived endocrine-disrupting chemicals (EDCs), among which bisphenol A (BPA; 2,2-bis(4-hydroxyphenyl)propane) is the most widely studied, are globally pervasive contaminants of aquatic ecosystems [1,2,3]. BPA has been repeatedly detected in surface waters at concentrations ranging from 170 to 3113 ng/L [3] and from <0.03 ng/L to 588,000 ng/L in European rivers [4], levels considered environmentally relevant for sub-chronic exposure. Because of its extensive industrial use and environmental persistence, BPA represents one of the major ecotoxicological concerns for aquatic organisms and humans [5].

The liver is a primary target of BPA toxicity, as it plays a central role in xenobiotic biotransformation and lipid homeostasis [6]. Numerous studies have demonstrated that even low and environmentally relevant concentrations of BPA induce hepatic steatosis, oxidative stress, mitochondrial dysfunction and lipid dysregulation in vertebrate models [7,8,9,10,11,12,13].

Adult zebrafish (*Danio rerio*) have emerged as a robust vertebrate model organism for investigating the mechanisms of hepatic toxicity [14,15,16,17]. Their liver shares its conserved histological architecture and lipid metabolic pathways with mammals, enabling the translation of biochemical, histological and omics-based data to humans [18,19].

Given the widespread presence of pollutants in the environment and the challenges in avoiding harmful contaminants in day-to-day life, probiotic supplementation has emerged as a promising tool to counteract metabolic toxicity and oxidative stress. Probiotics can modulate the gut microbiota, improve antioxidant capacity, and restore host metabolic balance through the gut–liver axis [20]. In a previous study carried out in our laboratory, a multi-strain probiotic formulation containing selected *Lactobacillus* and *Bifidobacterium* species showed mitigating effects against several environmental stressors in zebrafish [21,22,23]. Notably, growing evidence indicates that probiotic supplementation can mitigate the adverse effects of different environmental contaminants. Dietary administration of the probiotic formulation during sub-chronic exposure to environmentally relevant levels of BPA restored gut microbiota homeostasis, improved intestinal morphology, reduced hepatic steatosis and minimized oxidative damage along the gut–liver–brain axis [22], as well as attenuating BPA-induced reproductive alterations [23]. Consistently, *Bifidobacterium breve* and *Lactobacillus casei* were shown to decrease intestinal absorption of orally administered BPA in rats [24], whereas in zebrafish, *L. rhamnosus* counteracted the metabolic and neurotoxic effects of perfluorobutanesulfonate [25,26,27,28], and *L. plantarum ST-III* reduced triclosan-induced proinflammatory responses [29]. All these findings provide further evidence that probiotic administration can attenuate BPA-induced hepatotoxicity by modulating metabolic, inflammatory and apoptotic pathways. Nevertheless, to date, no studies have examined the ability of probiotic to mitigate BPA-induced alterations in liver lipid and carbohydrate metabolism.

Accordingly, in the present study, adult female zebrafish were exposed to an environmentally relevant concentration of BPA, to probiotics alone, or to BPA via the water and probiotic via the diet. Considering sex-dependent effects of BPA, as well as differences in the ability of probiotics to counteract BPA toxicity in reproduction [23] or metabolism [22], both processes strictly linked to lipid metabolism, females were exclusively employed in this study for more controlled and interpretable results. Given the complexity of these responses, a multidisciplinary analytical approach was essential to elucidate pathway-level perturbations. High-resolution lipidomics can provide detailed characterization of lipid class composition and molecular-species alterations that precede visible histological changes [10,30,31]. Complementarily, Fourier Transformed Infrared Imaging (FTIRI) spectroscopy allows us to obtain a label-free and rapid biochemical fingerprint of tissues, capturing global alterations in the macromolecular composition [32,33]. A combined use of these techniques can resolve, with a high degree of sensitivity, the biochemical and molecular endpoints of hepatic dysfunction.

The leading hypothesis of the present study is that sub-chronic exposure to low BPA doses can disrupt hepatic lipid homeostasis and carbohydrate/glycogen metabolism. Based on previous findings, we further hypothesized that probiotics could attenuate the effects of BPA exposure on hepatic lipid and carbohydrate metabolism in female zebrafish. Our integrative approach will provide insight into BPA-induced hepatic disruption and evaluate probiotic supplementation as an effective mitigation strategy to reduce the adverse actions of this contaminant.

## 2. Results

### 2.1. Hepatic Lipidomic Analysis

#### 2.1.1. Lipidomic Characterization of Liver Through PLS-DA Analysis

**Figure 1:**
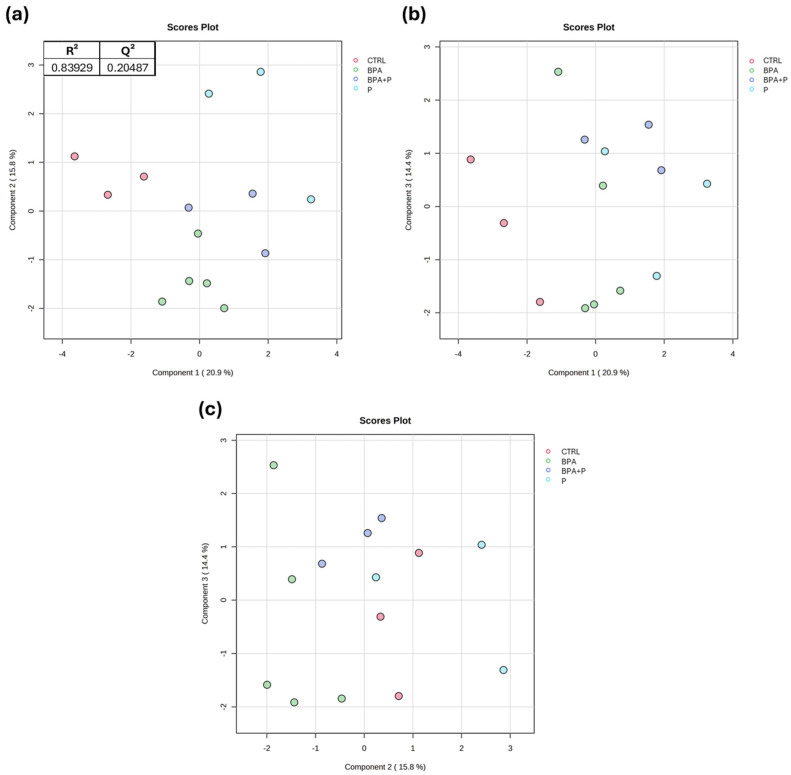
PLS-DA analysis of all groups. (**a**) PC1 vs. PC2, (**b**) PC1 vs. PC3 and (**c**) PC2 vs. PC3 (*n* = 3 for CTRL; *n* = 5 for BPA; *n* = 3 for BPA+P; *n* = 3 for P) with CTRL (red), BPA (green), BPA+P (blue), and P (light blue). Each symbol in the PLS-DA represents a pool of at least 3 livers. R^2^ and Q^2^ indicate the quality of the built models reported for each tested comparison.

Liver multivariate lipidomic analysis was conducted on all the experimental groups, building up three Partial Least Square-Discriminant Analysis (PLS-DA) models using Principal Component (PC) 1 and PC2, PC1 and PC3, and PC2 and PC3, respectively. The models revealed that PC1 clusters CTRL separately while partially clustering BPA+P and P together and separated from BPA (Figure 1a,b). PC2 clustered CTRL, BPA+P and P together with BPA partially overlapping with the BPA+P group (Figure 1a,c). In contrast, PC3 did not fully cluster the four experimental groups separately (Figure 1b,c). Taken together, these results indicate that the phenotype of the BPA+P group is intermediate between those of the BPA and P groups.

**Figure 2:**
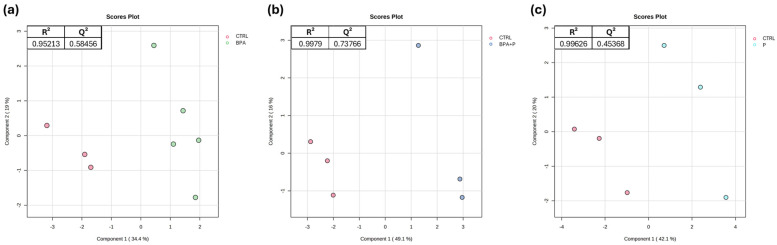
PLS-DA analysis of all groups. (**a**) CTRL vs. BPA, (**b**) CTRL vs. BPA+P and (**c**) CTRL vs. P (*n* = 3 for CTRL; *n* = 5 for BPA; *n* = 3 for BPA+P; *n* = 3 for P) with CTRL (red), BPA (green), BPA+P (blue), and P (light blue). Each symbol in the PLS-DA represents a pool of at least 3 livers. R^2^ and Q^2^ indicate the quality of the built models reported for each tested comparison.

Building up the PLS-DA models for the comparisons CTRL vs. BPA, CTRL vs. BPA+P, and CTRL vs. P, a clear separation was evident between CTRL and all the other experimental groups along PC1 (Figure 2a–c).

#### 2.1.2. Differential Lipid Analysis Through Volcano Plots

**Figure 3:**
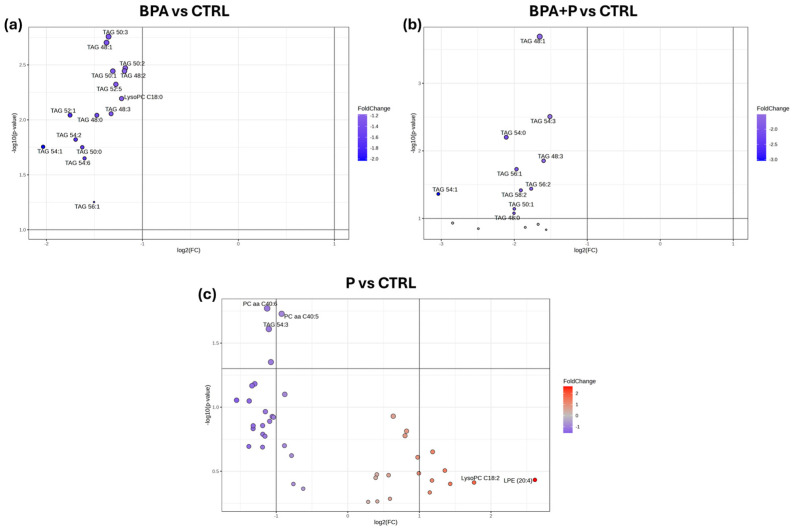
Volcano plot analysis. (**a**) BPA vs. CTRL, (**b**) BPA+P vs. CTRL, and (**c**) P vs. CTRL. Increased metabolites are shown in red (not statistically significant); downregulated metabolites are shown in blue (statistically significant).

The liver univariate lipidomic analysis was conducted using Volcano plots on the Variable Importance in Projection (VIP) values > 1 that emerged from the PLS-DA built for each comparison: CTRL vs. BPA, CTRL vs. BPA+P, and CTRL vs. P. BPA exposure resulted in the downregulation of 14 lipids (TAG 50:3, TAG 48:1, TAG 50:2, TAG 48:2, TAG 50:1, TAG 52:5, LysoPC C 18:0, TAG 48:3, TAG 52:1, TAG 48:0, TAG 54:2, TAG 54:1, TAG 50:0 and TAG 54:6) compared to CTRL (Figure 3a). Co-exposure to BPA and probiotic reduced the amount of eight lipids (TAG 48:1, TAG 54:3, TAG 54:0, TAG 48:3, TAG 56:1, TAG 56:2, TAG 58:2 and TAG 54:1) compared to CTRL (Figure 3b). The administration of the probiotic alone caused a reduction in three lipids (PC aa C40:6, TAG 54:3 and TAG 54:4) compared to CTRL (Figure 3c). Differentially Abundant Lipids Identified by Volcano Plot are reported in Table 1. 

### 2.2. FTIRI Analysis of Liver Samples

**Figure 4:**
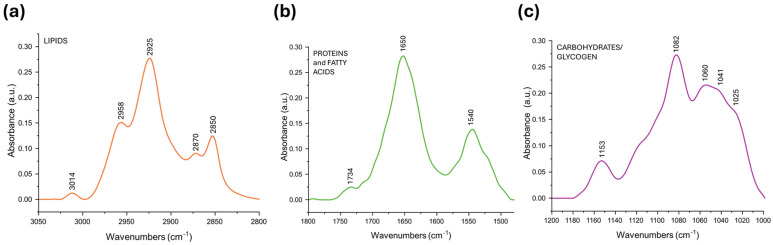
Representative IR spectrum of a liver sample from CTRL zebrafish female. The spectrum is displayed in absorbance mode in the following spectral intervals: (**a**) 3050-2800 cm^−1^, (**b**) 1800-1480 cm^−1^, and (**c**) 1200-1000 cm^−1^ ranges, corresponding, respectively, to the characteristic vibrational modes of lipids and fatty acids, proteins, and glycosylated compounds.

The IR absorbance spectrum of a representative liver section from CTRL female zebrafish is shown in Figure 4: three spectral intervals are displayed, representative, respectively, of lipids and fatty acids (3050-2800 cm^−1^; Figure 4a), proteins (1800-1480 cm^−1^; Figure 4b), and glycosylated compounds (1200-1000 cm^−1^; Figure 4c). The position of the main absorption peaks is summarized in Table 2, together with their spectral and biological assignments.

The hyperspectral imaging analysis was exploited on the spectral populations of CTRL, BPA, BPA+P, and P groups to explore the intrinsic variability in lipids and glycosylated compounds within the data set. The results are summarized below.

**Figure 5:**
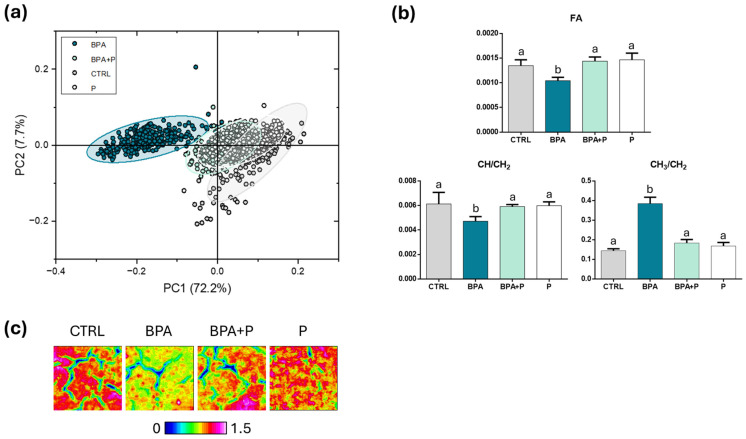
Hyperspectral imaging analysis of the lipid component. (**a**) PC score plot calculated on the spectral populations of CTRL, BPA, BPA+P and P groups (3050-2800 cm^−1^). (**b**) Statistical analysis of specific spectral parameters for lipids: FA (ratio between the integrated area of the peak at ~1734 cm^−1^ and the sum of the integrated areas of the regions 3050-2800 cm^−1^ and 1800-900 cm^−1^, representative of the total amount of fatty acids); CH/CH_2_ (ratio between the integrated areas of the peaks at ~3014 cm^−1^ and ~2924 cm^−1^, corresponding, respectively, to =CH and CH_2_ moieties in lipid alkyl chains, representative of the unsaturation degree of alkyl chains), and CH_3_/CH_2_ (ratio between the integrated areas of the peaks at ~2958 cm^−1^ and ~2924 cm^−1^, corresponding, respectively, to CH_3_ and CH_2_ moieties in lipid alkyl chains, representative of alkyl chain length). Data are normally distributed and presented as mean ± S.D. (*n* = 3 fish for each experimental group); different lowercase letters over histograms indicate statistically significant differences among groups (one-way ANOVA and Tukey’s multiple comparison tests; *p* < 0.05). (**c**) Representative false color images describing the topographical distribution and relative amount of fatty acids among the above cited experimental groups (integration of the IR images under the 3050-2800 cm^−1^ interval; dimension 164 × 164 µm^2^; 4096 pixel/spectra with a spatial resolution of 2.56 × 2.56 µm^2^). An arbitrary color scale was used, ranging from 0 (blue tones) to 1.5 (pink/white tones).

*Lipids and fatty acids*: As regards the lipid component, the PC score plot corresponding to the 3050–2800 cm^–1^ region evidenced a good separation between BPA and all other groups, which, on the contrary, almost overlapped (with explained variances along PC1 axis of 72.2% and along PC2 of 7.7%) (Figure 5a). The statistical analysis of the specific spectral parameters for characterizing the lipid composition (Figure 5b) highlighted the lowest amount of fatty acids (FA; *p* < 0.05) and CH moieties (CH/CH_2_; *p* < 0.05) and the highest one of CH_3_ groups (CH_3_/CH_2_; *p* < 0.05) in BPA, while all the other groups (CTRL, P and BPA+P) displayed similar values (*p* > 0.05). The false color images in Figure 5c reveal an almost homogeneous distribution of fatty acids in CTRL and P groups, while a more spotted localization was observed in BPA+P, and BPA, with this latter also showing the lowest abundance of lipids.

**Figure 6:**
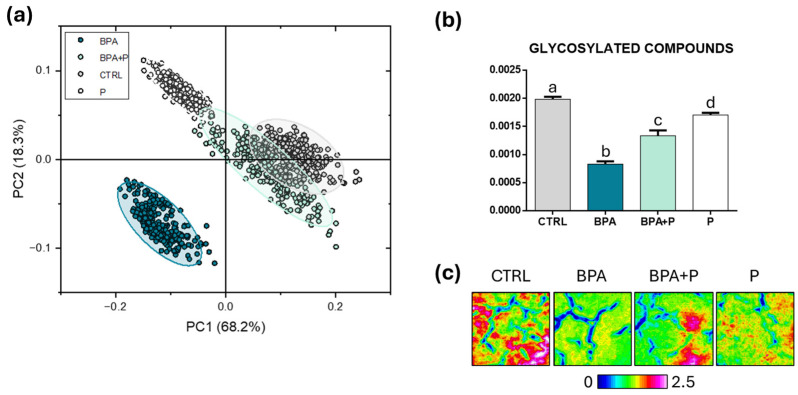
Hyperspectral imaging analysis of glycosylated compounds. (**a**) PC score plot calculated from IR spectra of CTRL, BPA, BPA+P and P experimental groups (1200-1000 cm^−1^). (**b**) Statistical analysis of the spectral parameter GLYCOSYLATED COMPOUNDS (ratio between the integrated area of the peak at ~1160 cm^−1^ and the sum of the integrated areas of the regions 3050-2800 cm^−1^ and 1800-900 cm^−1^, representative of the total amount of glycosylated compounds). Data are normally distributed and presented as mean ± S.D. (*n* = 3 fish for each experimental group); different lowercase letters over histograms indicate statistically significant differences among groups (one-way ANOVA and Tukey’s multiple comparison tests; *p* < 0.05). (**c**) Representative false color images describing the topographical distribution and relative amount of glycosylated compounds among the above cited experimental groups (integration of the IR images under the 1200-1000 cm^−1^ interval; dimension 164 × 164 µm^2^; 4096 pixel/spectra with a spatial resolution of 2.56 × 2.56 µm^2^). An arbitrary color scale was used, ranging from 0 (blue tones) to 2.5 (pink/white tones).

*Glycosylated Compounds*: As regards the spectral region 1200–1000 cm^−1^ (representative of glycosylated compounds), the PC scores plot highlighted a good separation of BPA spectra with respect to the CTRL and BPA+P ones along the PC1 axis (explained variance, 68.2%) and to P ones along the PC2 axis (explained variance 18.3%); moreover, CTRL and BPA+P were almost segregated with respect to P along the PC1 axis (explained variance, 68.2%) (Figure 6a). The statistical analysis of the spectral parameter GLYCOSYLATED COMPOUNDS indicated that BPA presents the lowest value and CTRL the highest one (*p* < 0.05), while BPA+P and P groups display intermediate values (*p* > 0.05) (Figure 6b). These findings are also confirmed by the false color images in Figure 6c, which indicate the lowest amount of these biomolecules in BPA.

## 3. Discussion

In this study, the toxic effects of sub-chronic exposure to a low BPA dose and the concomitant mitigation due to probiotic administration were investigated in female zebrafish, using an integrated lipidomic and FTIRI approach. It is worth noting that, while BPA-induced hepatic steatosis has been widely reported in the literature [7,19,36,37], available evidence suggests that these effects are highly dependent on exposure dose and experimental context [12]. In this regard, in accordance with our results, it was recently observed that a hepatic lipid depletion, rather than an accumulation, occurred in female zebrafish exposed to a high concentration of BPA (5 mg/L) coupled with a reduction in ceramides, fatty acids, lysoglycerophosphoinositols, glycerophosphoethanolamines, and glycerophosphoinositols [10]. In this light, the results suggest that BPA behaves in a dose-dependent manner and that the exposure can differently affect hepatic lipid metabolism. The results herein presented demonstrated that the exposure to BPA markedly decreased multiple triacylglycerol species spanning a range of carbon chain lengths and degrees of unsaturation, mainly unsaturated TAGs, an alteration in agreement with previously observations in a zebrafish liver cell line exposed to 50 µM of BPA [9]. These data are also consistent with what previously observed in our laboratory by exposing zebrafish female to BPA for 21 days, at the same BPA dose used in the present study, finding a fatty acid decrease [12]. TAGs serve as the primary storage form of neutral lipids in hepatocytes and function as reservoirs of energy and fatty acids for membrane biosynthesis [38]. Males are more protected than females from developing liver steatosis, and this is also true in the presence of EDCs like BPA [39]. Indeed, the study by Tassinari et al. [13], who exposed rats to BPA doses similar to those measured in the Italian population, showed that while male rats showed increased liver size, this did not occur in the liver of female rats. BPA-induced TAG depletion is likely to be mediated by the activation of estrogen receptors [40], which in the liver has been shown to suppress de novo lipogenesis (DNL), enhance fatty acid β-oxidation, and improve insulin sensitivity [41]. This is consistent with known effects of various xenobiotics, which disrupt hepatic lipid metabolism by interfering with nuclear receptor signaling and lipid balance, while also promoting oxidative stress and lipid peroxidation [42,43,44,45,46,47]. FTIRI analysis supported and expanded these lipidomic observations, as suggested by the decrease in fatty-acid spectral parameter (FA) upon exposure to BPA. In addition, the decrease in the CH/CH_2_ spectral parameter and the increase in the CH_3_/CH_2_ one suggest the presence of shorter lipid alkyl chains with a higher saturation degree.

In parallel, BPA exposure reduced the levels of lysophosphatidylcholine C18:0. LysoPCs are important intermediates in membrane remodeling and participate in inflammatory signaling processes [48]. Their depletion in hepatic tissue is thus indicative of possible disrupted membrane turnover and potential impairment of intracellular signaling. Bisphenols have been associated with altered phospholipid metabolism, where reduced LysoPC and phosphatidylcholine levels correlate with oxidative damage and compromised membrane integrity [49,50,51]. These disruptions are particularly relevant given that phospholipid balance is crucial for membrane fluidity, vesicle formation, and lipid droplet synthesis, all of which are essential for normal hepatocyte functions. In addition, the reduction in lysophosphatidylcholine C18:0 together with decreased triglyceride levels following BPA exposure is consistent with estrogen receptor-dependent metabolic reprogramming in the liver. Estrogenic signaling suppresses triglyceride synthesis while promoting fatty acid oxidation and phospholipid remodeling [52], leading to reduced TG storage and diminished LPC pools through enhanced re-acylation. This profile reflects altered lipid partitioning rather than overt steatosis and may represent an early or exposure-specific metabolic phenotype relevant to MASLD.

FTIRI also revealed a pronounced depletion of hepatic glycosylated compounds (ascribable also to glycogen) in BPA-exposed females, an observation in accordance with previous results regarding the effects of this BPA concentration on female zebrafish livers [22]. Glycogen is the principal storage form of glucose in the liver, and its reduction reflects altered energy homeostasis, increased metabolic demand, and disruption of normal carbohydrate metabolism [53,54]. BPA has been shown to interfere with glucose-regulatory pathways, including modulation of glycolytic and gluconeogenic gene expression, leading to impaired glucose storage and utilization [55]. This depletion of glycosylated compounds, likely ascribable to glycogen reserves, given the biological nature of the liver as a primary site of glycogen accumulation, together with the lipid losses observed in lipidomic and FTIR analyses, suggests that BPA exposure shifts metabolic flux toward increased energy mobilization, reflecting a stress response that consumes readily available and stored energy reserves.

Interestingly, in the BPA+P co-exposed fish, the multi-strain probiotic mix mitigated many of the BPA-induced biochemical alterations: a lower number of TAG species were reduced, and no detectable perturbation of fatty acids and glycosylated compounds was registered. Probiotics can modulate intestinal barrier integrity, limit BPA absorption, and promote microbial biotransformation or sequestration of EDCs [56,57,58], thereby attenuating estrogen receptor-mediated hepatic metabolic effects [59,60]. In parallel, probiotic-induced normalization of gut–liver signaling, including bile acid composition, short-chain fatty acid production, and inflammatory tone, may stabilize hepatic lipid, protein, and carbohydrate metabolism [61]. The persistence of only a limited number of reduced TAG species suggests partial protection, consistent with dampened endocrine and metabolic stress rather than complete reversal of BPA exposure effects.

Differently from BPA alone, probiotic co-administration reduced the levels of TAG 54:0, a saturated TAG, and TAG 54:3, which has been previously identified as one of the TAG species increased in the plasma and liver of C57Bl/6J mice fed a high-fat, high-sucrose diet [62] and associated with metabolic dysfunction and associated steatosis liver disease (MASLD) phenotypes and metabolic stress in rodent models [63], suggesting a hepatoprotective role. Thus, the observed reduction in TAG 54:0/54:3 in the BPA+P group suggests an increase in the degree of unsaturation of hepatic fatty acids, at the expense of saturated fatty acids. Saturated fatty acids are known to promote lipotoxicity and contribute to hepatic steatosis, whereas more unsaturated fatty acids are generally considered hepatoprotective [64]. These findings are in line with previous evidence that the probiotic mix exerts a hepatoprotective effect under conditions of sub-chronic BPA exposure [22]. The effects of the probiotic mixture alone, in fact, were distinct from those of BPA and characterized by selective decreases in certain polar lipids and highly unsaturated TAGs, patterns more consistent with regulated membrane lipid remodeling rather than broad metabolic dysfunction. Probiotic interventions have been shown to interact with host lipid metabolism and modulate lipidomic profiles, possibly by influencing gut microbiota composition and metabolic pathways linked to glycerophospholipid and fatty acid metabolism [65]. Moreover, the presence and activity of specific gut microbes have been associated with alterations in phosphatidylcholine and related lipid species, indicating that microbiota-mediated regulation can selectively affect lipid classes [66,67,68]. In this light, we previously observed that the administration of SLAB51 increased *Cetobacterium* in BPA+P females’ intestines [22]. This bacterium is part of the fish gut microbiota and it is known to produce acetate, a short-chain fatty acid (SCFA) with systemic signaling roles [69]. Enrichment of *C. somerae* has been causally linked to improved glucose homeostasis and increased insulin expression in zebrafish, likely mediated via an acetate-dependent gut–brain axis and associated with better host metabolic regulation [69]. SCFAs such as acetate, propionate, and butyrate produced by gut microbes not only serve as energy substrates but also engage host signaling pathways involved in lipid and glucose metabolism, inflammation, and liver function via GPCRs and other metabolic regulators [70]. Thus, the probiotic-induced enrichment of *Cetobacterium* may contribute to hepatoprotective effects by influencing gut microbiota-derived metabolite pools that support energy homeostasis, modulate lipid processing, and attenuate metabolic stress in the host.

Mechanistically, probiotics like the one selected in the present study have been shown to modulate host metabolism, enhance antioxidant defenses, and improve intestinal barrier integrity. Probiotic supplementation has been associated with the modulation of both metabolic parameters and increased antioxidant capacity in clinical and preclinical studies [71], improvement of gut barrier function and reduction in inflammation [72], and influencing host metabolic health via gut microbiota modulation [73]. The selected probiotic mix has demonstrated the capacity to reduce oxidative stress through activation of antioxidant pathways which can stabilize hepatic lipid metabolism and reduce peroxidative damage [74]. Additionally, this probiotic mix has been reported to normalize aspects of the gut microbiota disrupted by BPA and to restore gut–liver communication, thereby reducing systemic inflammation and hepatic metabolic stress [22]. Probiotic modulation of the gut microbiome may also influence enterohepatic signaling and bile acid metabolism, which are known regulators of lipid and carbohydrate homeostasis [75,76,77]. All these positive effects of the probiotic mix could have contributed to the mitigation capacity of this probiotic against BPA sub-chronic exposure hepatotoxicity observed in the present study.

While the present findings strongly support a protective role for the probiotic mixture, the underlying molecular pathways remain to be fully elucidated. In the future, it may be useful to further investigate oxidative stress markers (e.g., lipid peroxidation products, ROS levels) and mitochondrial function assays, which could provide additional insights into the potential links between metabolic regulation and the protective effects of probiotics against EDC-induced liver alterations. A limitation of the present study is that livers were pooled from individuals across the two replicate tanks per treatment to obtain sufficient tissue for the analyses. This pooling likely reduced the variability in the measurements and limited the statistical inference that can be drawn at the individual level. As a result, the data should be considered semi-quantitative rather than fully quantitative. Despite this, the approach allowed identification of specific lipid species whose levels may be influenced by the treatments, providing valuable mechanistic insights while acknowledging the constraints on statistical interpretation.

## 4. Materials and Methods

### 4.1. Probiotic Mix SLAB51

The commercial probiotic SLAB51 (SivoMixx^®^, Ormendes SA, Jouxtens-Mézery, Switzerland) is a multi-strain formulation that includes eight lyophilized bacterial strains: *Streptococcus thermophilus DSM 32245* (80 billion CFU); *Bifidobacterium lactis DSM 32246* (25 billion CFU); *Bifidobacterium lactis DSM 32247* (25 billion CFU); *Lactobacillus acidophilus DSM 32241* (5 billion CFU); *Lactobacillus helveticus DSM 32242* (1 billion CFU); *Lactobacillus paracasei DSM 32243* (12 billion CFU); *Lactobacillus plantarum DSM 32244* (16 billion CFU); and *Lactobacillus brevis DSM 27961* (36 billion CFU), with a total bacterial concentration of 2 × 10^11^ CFU/g.

### 4.2. Fish Maintenance and Experimental Design

A total of 80 healthy six-month-old female wild-type zebrafish (AB strain, *Danio rerio*), all of similar size and free from abnormalities, were housed in glass tanks with oxygenated water. They were kept under standard conditions with a temperature of 28.0 ± 0.5 °C and a 14/10-h light/dark cycle. The water chemical and physical parameters were continuously monitored. The zebrafish were fed daily with dry food (TetraMin Granules, Tetra, Melle, Germany). The daily feed was administered twice per day at a total amount equal to 3% of body weight. The two feedings were distributed as follows: the first portion, corresponding to 1/10 of the total daily ration, was provided at 9:00 a.m., and the second portion, corresponding to the remaining 9/10, was provided at 5:00 p.m. The probiotic was administered during the first feeding to ensure complete consumption within a short period of time (1–2 min). The experiment was conducted in duplicate, using 30 L glass tanks in a semi-static system. The adult zebrafish were divided into four groups, each with 20 females divided into 2 glass tanks housing 10 fish each per group. The experiment was then conducted in biological duplicate with both tanks per group running simultaneously under identical environmental conditions within the same facility, with identical tanks and time frame. Excess food and feces were removed daily by siphoning the bottom of the tank, followed by a water top-up. The water quality was monitored using water tests for pH and nitrates, and temperature was strictly controlled. The control group (CTRL) received commercial dry food; the probiotic group (P) was fed dry food supplemented with lyophilized SLAB51 at a concentration of 10^9^ CFU/g of body weight; the BPA group was fed commercial dry food and exposed to 10 μg/L BPA (98% analytical purity, Sigma-Aldrich, Milan, Italy) in water; and the BPA + SLAB51 (BPA+P) group was fed dry food enriched with SLAB51 at a concentration of 10^9^ CFU/g of body weight and exposed to 10 μg/L BPA in water. The BPA concentration was chosen based on its documented effects on liver metabolism [12,22], while the SLAB51 concentration was based on prior research in mice [78,79] and zebrafish [21,22,23]. The experiment was performed following the University of Calgary’s animal care protocol (AC24-0042 approved in date 1 May 2025) to ensure ethical treatment of the animals, and all efforts were made to minimize suffering. No deaths occurred in any of the experimental groups during the exposure. After 28 days, the fish were humanely euthanized with an overdose of MS-222 (3-aminobenzoic acid ethyl ester, Merck, Darmstadt, Germany), buffered to pH 7.4, as per the University of Calgary’s guidelines. Liver samples were collected and stored at −80 °C for further analysis.

### 4.3. Lipid Extraction and UHPLC-QTOF-MS Analysis

Lipidomic analysis was performed on pools of liver tissue formed by combining individuals from both tanks within the same experimental group in a balanced manner, in order to avoid overrepresentation of a single tank, despite the exact number of livers per each pool from the two tanks not being recorded. The pooling approach was adopted to ensure sufficient material for reliable lipidomic profiling. Liver weight guided the number of pools that were possible to obtain for this analysis. Lipid species were extracted from homogenized liver samples with a modified Folch method: 600 µL of chloroform:methanol (2:1) and 100 µL of water. The lower phases were dried under a gentle nitrogen flux and samples were reconstituted in 100 µL methanol:chloroform (95:5). Lipidomic analysis was performed using liquid chromatography/quadrupole time-of-flight mass spectrometry (UHPLC-QTOF, 1290 Infinity-6540 Agilent Technology, Santa Clara, CA, USA) with an electrospray ionization source (ESI) (Agilent Technology, Santa Clara, CA, USA) equipped with an Agilent ZORBAX Eclipse Plus C18 2.1 × 100 mm 1.8-Micron column (Agilent Technology, Santa Clara, CA, USA) at 50 °C. The mobile phase A was water with 0.1% formic acid and the mobile phase B was isopropanol/acetonitrile (1:1, *v*:*v*) with 0.1% formic acid. The injection volume was 1 µL. The flow rate was 0.4 mL/min for the first 9 min and 0.6 mL/min for the last 7 min. The gradient used for lipid separation was as follows: 0 min 35% B; 0–2 min 80% B; 2–9 min 100% B; 9–16 min 100% B. The post-run was set at 6 min. Acquisition was in positive mode and the acquisition rate was 2 spectra/s, mass range 100–1200 m/z, capillary voltage 3500 V, nozzle voltage 1000 V, gas temperature 200 °C, drying gas of nitrogen at a flux of 8 L/min, nebulizer gas (nitrogen) 35 psi, sheath gas temperature 350 °C, and sheath gas flow (nitrogen) of12 L/min. The injection volume was 1 µL. Continuous mass calibration was performed by monitoring reference ions at m/z 121.0509 m/z and 922.0098 m/z (purine and HP-0321Agilent) to correct the mass assignments during a sample run and to maintain high mass measurement accuracy. Liver samples were analyzed in MS mode, solvent blanks were injected regularly (two at the beginning of the sequence, two every ten samples, and two at the end of the sequence) and NIST SRM 1950 (Metabolites in Frozen Human Plasma) reference material was used to control the stability of the method. The analysis of the spectra was performed using the Agilent MassHunter Profinder B.08.00, a mass spectrometry-based algorithm batch-targeted feature extraction software. Lipid identifications were matched against our custom in-house lipid library, constructed in Agilent PCDL B.08.00 and validated with targeted lipids. Spectrum integration was manually curated. Match tolerance for masses was set at 10 ppm and the isotopic distribution (abundance and spacing) was used to confirm lipid identity and charge state. Retention time alignment was performed with a ±0.1 min window, and a minimum peak intensity of ≥1000 counts was required for detection.

Lipid concentrations were quantified with internal standards added to each sample, i.e., TAG (15:0/15:0/15:0), DAG (17:0/17:0), PC (17:0/17:0), PE (17:0/17:0), LPC (17:0), LPE (17:1), CER (d18:1/17:0), purchased from Avanti Polar Lipids (Alabaster, AL, USA) and Larodan (Solna, SE). Lipids were quantified as [M+H]^+^ adducts for PCs, PEs, LPCs, and CERs and as [M+Na]^+^ adducts for TAG and DAG.

### 4.4. FTIRI Analysis

Infrared imaging analysis was carried out using an INVENIO-R interferometer combined with a Hyperion 3000 Vis-IR microscope and equipped with a Focal Plane Array (FPA) detector cooled with liquid nitrogen (Bruker Optics, Ettlingen, Germany). The use of the FPA detector enables the acquisition of IR images on non-homogeneous biological materials, such as tissues; each IR image covered an area of 164 × 164 µm^2^, consisting of 4096 individual pixels/spectra, with a spatial resolution of 2.56 × 2.56 µm^2^/pixel. This imaging analysis allows the correlation of morphological and chemical information within the same field of view.

The following procedure was followed. Liver samples (*n* = 3 for each experimental group) were embedded in Killik O.C.T. (Bio Optica, Milan, Italy). From every specimen, two sections were cut with a thickness of 8 µm (MC4000 cryostat, Histo-Line, Milan, Italy, maintained at –26 °C). Sections were deposited onto CaF_2_ optical windows (1 mm thick, 13 mm diameter) and air-dried for 30 min before IR analysis. On each section, an IR image was recorded in transmission mode over the 4000-900 cm^−1^ spectral range, with a spectral resolution of 4 cm^−1^; every spectrum was obtained by averaging 256 scans. Before each image acquisition, a background spectrum was collected on a clean region of the CaF_2_ optical window. Raw data were first processed to remove contributions from atmospheric CO_2_ and water vapor and to minimize spectral alterations due to variations in section thickness (Atmospheric Compensation and Vector Normalization routines (OPUS 7.1 software, Bruker Optics).

Pre-processed IR images thus obtained were then integrated over the following spectral regions to generate false color images (size 164 × 164 µm^2^) illustrating the topographical distribution and relative abundance of the most relevant liver macromolecules: 3050-2800 cm^−1^ (representative of alkyl groups in lipids and fatty acids), 1800-1480 cm^−1^ (representative of proteins), and 1200-1000 cm^−1^ (representative of glycosylated compounds). An arbitrary color scale was adopted, with white/pink tones indicating areas of higher absorbance while blue ones represent lower absorbance levels (OPUS 7.1 software, Bruker Optics).

From each pre-processed IR image, ca. 50 IR spectra were extracted and then subjected in absorbance mode to Principal Component Analysis (PCA) in the 3050-2800 cm^−1^, and 1200-1000 cm^−1^ ranges upon vector normalization and two-point baseline correction (OPUS 7.1 for pre-processing and OriginPro 2023 for statistical computation).

Finally, all the IR spectra, extracted from the IR images, were integrated under specific spectral ranges (OPUS 7.1 software, Bruker Optics, Ettlingen, Germany), and the areas obtained were used to calculate the following spectral parameters: CH/CH_2_ (ratio between the integrated areas of the peaks at 3014 cm^−1^ and 2924 cm^−1^, corresponding, respectively, to =CH and CH_2_ moieties); CH_3_/CH_2_ (ratio between the integrated areas of the peaks at 2958 cm^−1^ and 2924 cm^−1^, corresponding, respectively, to CH_3_ and CH_2_ moieties); FA (ratio between the integrated area of the peak at 1734 cm^−1^, representative of the total absorbance of fatty acids, and the sum of the integrated areas of the regions 3050-2800 cm^−1^ and 1800-900 cm^−1^, representative of the total absorbance of the sample); and GLYCOSYLATED COMPOUNDS (calculated as the ratio between the integrated area of the peak at 1160 cm^−1^, representative of the total absorbance of C-OH groups in glycosylated compounds, and the sum of the integrated areas of the regions 3050-2800 cm^−1^ and 1800-900 cm^−1^, representative of the total absorbance of the sample).

### 4.5. Statistical Analysis

The MetaboAnalyst 5.0 online platform (University of Alberta, Alberta, Canada, accession date 24 October 2025) was used to perform PLS-DA [21,22,80]. Univariate analysis was performed using Volcano plots on lipids with a Variable Importance in Projection (VIP) score > 1 in each model, using Metaboanalyst 5.0 as described previously [21,22,80]. A significant threshold of *p* value < 0.05 was used to assess statistically significant differences among experimental groups. Because exposure occurred at the tank level while analyses were conducted on pooled samples, tank effects cannot be included in the statistical models. As a result, the findings are best interpreted as semi-quantitative rather than fully quantitative, with limited resolution at both the individual and tank levels.

The spectral parameters, calculated as described in Section 4.4, were statistically analyzed as follows. First, we considered the data from each section: they were averaged upon verifying that they were normally distributed (Shapiro–Wilk normality test). Then, these mean values were averaged across the two sections, and the resulting average values were statistically analyzed by one-way ANOVA followed by Tukey’s multiple comparison test (*n* = 3 fish per experimental group). Statistical software package Prism 8 (GraphPad Software, Inc., San Diego, CA, USA) was used with significance accepted at a *p*-value < 0.05.

## 5. Conclusions

Sub-chronic exposure to a low, environmentally relevant concentration of BPA altered hepatic metabolism in female zebrafish by reducing TAG reserves, modifying fatty acid profiles, and markedly depleting glycosylated compounds. These findings demonstrate that BPA disrupts multiple metabolic pathways, affecting energetic components of hepatic function. Co-treatment with the probiotic mix mitigated many of these alterations by partially preserving triacylglycerol content, improving fatty acid structural integrity, and attenuating glycosylated compound loss. Overall, the results indicate that the probiotic mix may exert mitigating effects against BPA-induced metabolic disruption, supporting its potential as a nutritional strategy to reduce the hepatotoxicity associated with sub-chronic exposure to EDCs. Unfortunately, the need to use pooled samples did not allow us to model these data at the tank level, limiting statistical inference; therefore, these findings should be interpreted as semi-quantitative. However, these findings are relevant for regulatory agencies evaluating the health risks of sub-chronic, low-dose BPA exposure, highlighting the need for stricter safety guidelines. Moreover, the potential use of probiotics as a dietary intervention offers a practical, accessible approach for the general population to reduce the impact of environmental toxicants, bridging the gap between environmental health research and public health applications.

## Figures and Tables

**Table 1:** Differentially Abundant Lipids Identified by Volcano Plot (Fold Change and *p*-values).

(a) BPA vs. CTRL			(b) BPA+P vs. CTRL			(c) P vs. CTRL		
Name	Fold Change (FC)	*p*	Name	Fold Change (FC)	*p*	Name	Fold Change (FC)	*p*
TAG 50:3	0.39159	0.0017485	TAG 48:1	0.31837	0.000204	PC aa C40:6	0.45794	0.016943
TAG 48:1	0.38588	0.0019833	TAG 54:3	0.35069	0.003104	TAG 54:3	0.46546	0.024556
TAG 50:2	0.44185	0.0033781	TAG 54:0	0.23169	0.006287	TAG 54:4	0.47495	0.044476
TAG 48:2	0.4393	0.0035993	TAG 48:3	0.33033	0.014019	TAG 54:2	0.40612	0.065754
TAG 50:1	0.40379	0.0036009	TAG 56:1	0.25538	0.01866	TAG 56:4	0.3955	0.068011
TAG 52:5	0.41285	0.0047521	TAG 56:2	0.29359	0.036324	TAG 54:1	0.34057	0.088232
LysoPC C 18:0	0.43013	0.0064013	TAG 58:2	0.26619	0.038339	TAG 56:3	0.38491	0.089445
TAG 48:3	0.39878	0.0088014	TAG 54:1	0.17239	0.043383			
TAG 52:1	0.29648	0.0090641	TAG 50:1	0.37641	0.072102			
TAG 48:0	0.35979	0.0090837	TAG 48:0	0.35707	0.083785			
TAG 54:2	0.30864	0.015116						
TAG 54:1	0.24407	0.017589						
TAG 50:0	0.32387	0.017767						
TAG 54:6	0.32921	0.022407						
TAG 56:1	0.35256	0.055988						

**Table 2:** Position (wavenumber, cm^−1^), vibrational modes and biochemical interpretation of the most significant IR peaks, identified by second derivative minima analysis, in the IR spectrum of a representative liver section from CTRL female zebrafish [34,35].

Wavenumber (cm^−1^)	Vibrational Modes	Biochemical Assignments
~3014	Stretching vibration of =CH groups in alkyl chains	Lipids and fatty acids
~2958, ~2870	Asymmetric and symmetric stretching vibrations of CH_3_ groups in alkyl chains	
~2925, ~2850	Asymmetric and symmetric stretching vibrations of CH_2_ groups in alkyl chains	
~1734	Stretching vibration of C=O ester groups	
~1650, ~1540	Stretching vibrations of C=O and C-N groups and bending vibration of N-H groups (respectively, Amides I and II)	Proteins
~1082	Stretching vibration of PO_2_^–^ groups	Phosphate groups in nucleic acids
~1160, ~1060, ~1041, ~1025	Stretching vibration of C-C, C-O and C-OH groups	Glycosylated compounds

## Data Availability

The original contributions presented in this study are included in the article. Further inquiries can be directed to the corresponding authors.
